# A dataset on public participation intention in smart city development among Vietnamese citizens

**DOI:** 10.1016/j.dib.2026.113211

**Published:** 2026-08-30

**Authors:** Thi Thanh Hoa Phan, Ho Diem Chau Nguyen, Le Trung Duong, Thi Yen Nhi Pham, Minh Tam Bui, Viet Hoang Le, Van Trung Tran

**Affiliations:** Faculty of Business Management, National Economics University, 207 Giai Phong Street, Bach Mai Ward, Ha Noi City, Vietnam

**Keywords:** Smart city, Trust in the government, TAM

## Abstract

Our research presents a survey dataset on Vietnamese citizen's public participation intention in smart city development. The data was collected from residents living in three major urban areas in Viet Nam, namely Ha Noi, Da Nang, and Ho Chi Minh, which represent different contexts of smart city development. The survey was designed based on the smart city perception framework encompassing six dimensions such as Smart Economy, Smart People, Smart Governance, Smart Mobility, Smart Environment, and Smart Living combined with the Technology Acceptance Model (TAM), including its main components of perceived usefulness, perceived ease of use, and public participation intention in a smart city. In addition, trust in the new local government was implemented as a unique feature in the research model. Besides the theoretical constructs, the survey also collected respondents’ demographic information, such as gender, age, educational level, workplace, and area of residence to support control analyses and comparisons across population groups. A total of 846 valid responses were retained for analysis. The dataset provides valuable empirical evidence for research on smart cities, technology acceptance behavior, and is available for comparative analyses across population groups and urban contexts in Viet Nam.

Specification Table

**Table:** 

Subject	Urban Studies
Specific subject area	Smart City
Type of data	Table
How data was acquired	Questionnaires
Data format	Raw
Description of data collection	Data was collected from September 2025 to January 2026. Data was collected from residents in Ha Noi, Da Nang, and Ho Chi Minh using an online questionnaire. After screening the data, the final data set consisted of 846 observations.
Data source location	Region: Asia Country: Viet Nam
Data accessibility	The dataset is provided as a supplementary file. Data identification number: 10.17,632/2yfkrcy6d7.2 Direct URL to data: https://data.mendeley.com/datasets/2yfkrcy6d7/2

## 1 Value of Data

• The dataset can serve as a baseline for longitudinal studies assessing changes in citizens’ trust in newly restructured local governments and their acceptance of smart city development after the new governance system has been implemented over time.
• The dataset provided in this study can be used by future researchers to extend the proposed model by adding factors such as service quality, satisfaction, quality of life, privacy concerns, or perceived risk.
• In addition to theoretical constructs, the dataset includes respondents’ demographic information, such as place of residence, age, gender, educational level, and type of organization or sector of employment, allowing control of individual characteristics and analysis of perceptual and behavioral differences across population groups.
• The dataset can support comparative studies by enabling researchers to examine differences in smart city perceptions, acceptance, trust in local government, and public participation intention across Ha Noi, Da Nang, and Ho Chi Minh, as well as across demographic groups. It can also serve as a baseline for future studies in other Vietnamese cities or at different points in time.
• The dataset can provide an empirical basis for policy recommendations by helping local authorities identify smart city dimensions requiring greater attention and tailor development priorities to local contexts. Comparisons across cities and insights into citizens’ trust in local government can further support more targeted and citizen-oriented smart city policies.

## 2 Background

Rapid urbanization and digital transformation have increased the importance of Smart Cities as an approach to improving urban governance, public services, and citizens’ quality of life [1,2]. Among the widely adopted theoretical frameworks, the six pillar model proposed by Giffinger et al [3] including Smart Economy, Smart Mobility, Smart Environment, Smart Living, Smart People, and Smart Governance, which is regarded as a foundational framework for conceptualizing and measuring the development level of Smart Cities from a comprehensive perspective. Building on this definition, citizen perceptions are important for understanding how individuals respond to Smart City development, while trust in local government represents a relevant institutional factor in government and citizen interactions. However, empirical data documenting citizens’ perspectives on Smart City development remains limited in developing country contexts, particularly amid institutional and administrative reforms.

This dataset was compiled in Viet Nam to examine citizens’ perceptions of Smart City development and their responses within a changing urban governance context. The data was generated using a structured questionnaire based on established measurement scales and collected from citizens in major Vietnamese cities. These include measures capturing multidimensional Smart City perceptions, citizens’ responses toward Smart City development, and trust in the new local government. In relation to the associated research study, this article provides the underlying dataset, measurement items, and methodological information, enabling researchers to further examine, validate, or extend analyses of citizen-centric Smart City development in the Vietnamese context.

## 3 Data Description

The research sample was collected from residents living in three major cities in Viet Nam: Ha Noi, Da Nang, and Ho Chi Minh. Additionally, the team collected survey responses from residents by sending survey forms on social media platforms such as Facebook, Email and filling out forms directly on the devices of the team members. By clearly explaining the goals, objectives, and value of the research, residents in the three major cities voluntarily agreed to answer the survey and did not receive any remuneration to support the research. An online questionnaire was designed with two main sections: personal information and residents’ perceptions of smart city elements. The first section consists of demographic questions related to personal characteristics (such as gender, age, education level, workplace, etc.). This content is presented in detail in Table 1. The questions in the second section were designed based on the characteristics of smart cities according to the classification of Giffinger et al [3] and inherited and improved the questionnaire from the study of Pinochet et al. [4], Wang [5], Yu [6] and Ling et al. [7]. The results were analyzed based on 846 valid responses collected by the authors.

**Table 1:** Respondents’ characteristics.

Characteristics	N	%
***Area of Residence***	**846**	**100.00%**
Ha Noi	325	38.42%
Da Nang	199	23.52%
Ho Chi Minh	322	38.06%
***Gender***	**846**	**100.00%**
Male	345	40.78%
Female	493	58.27%
Other	8	0.95%
***Age***	**846**	**100.00%**
Under 18	64	7.57%
18–24	573	67.73%
25–35	92	10.87%
36–45	45	5.32%
Over 45	72	8.51%
***Education Level***	**846**	**100.00%**
High School or lower	102	12.06%
College/University	697	82.39%
Post-graduate (Master’s, PhD…)	31	3.66%
Other	16	1.89%

**Fig. 1:**
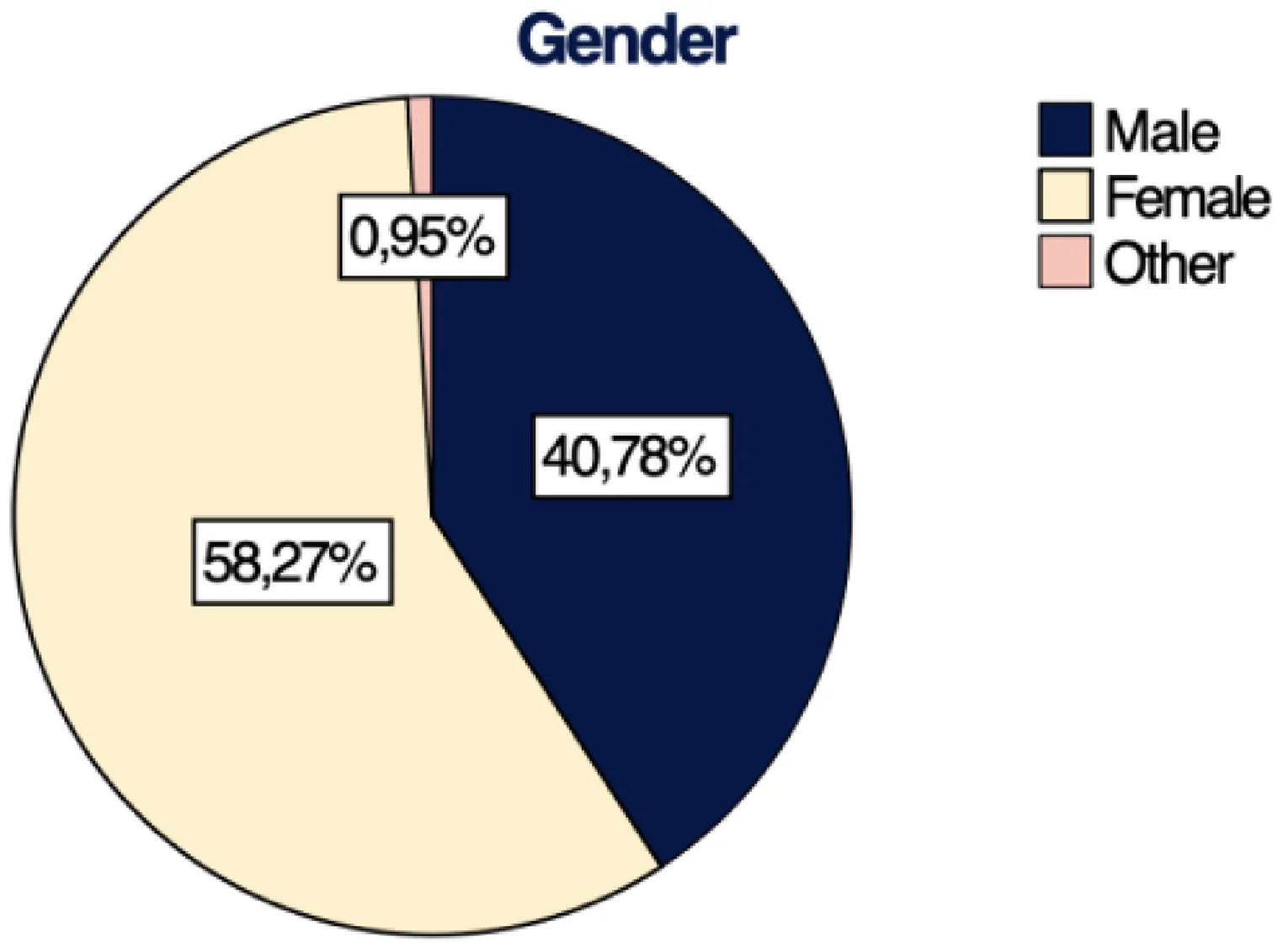
Gender distribution of respondents.

**Fig. 2:**
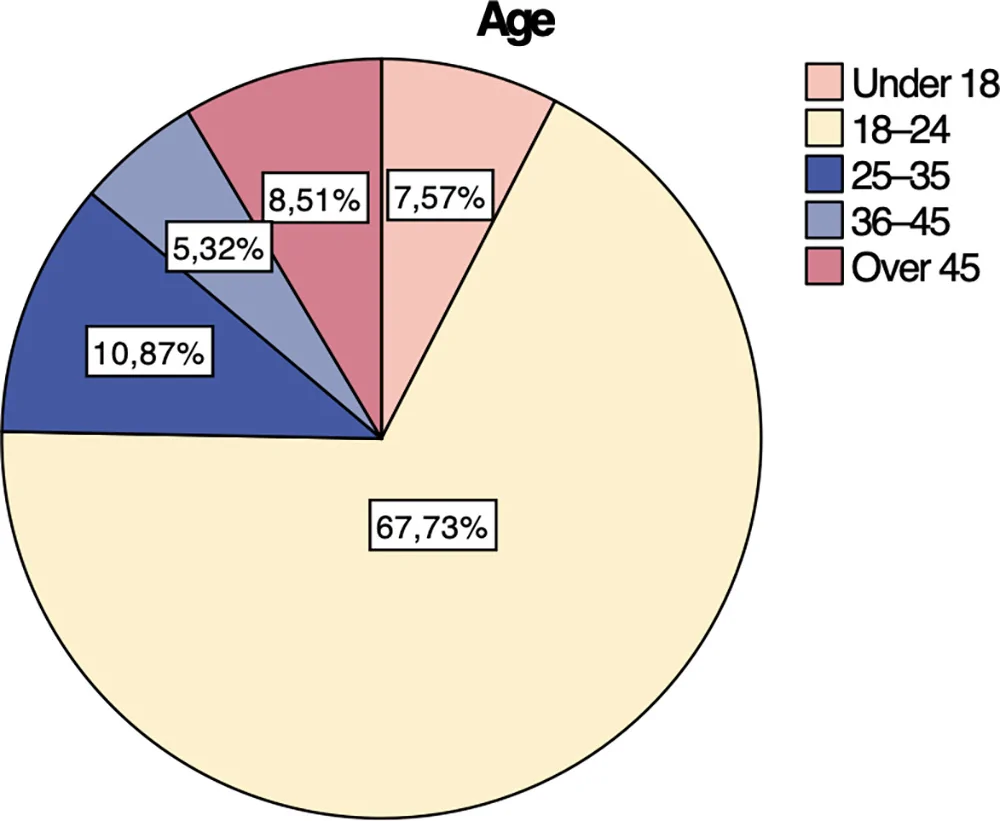
Age distribution of respondents.

**Fig. 3:**
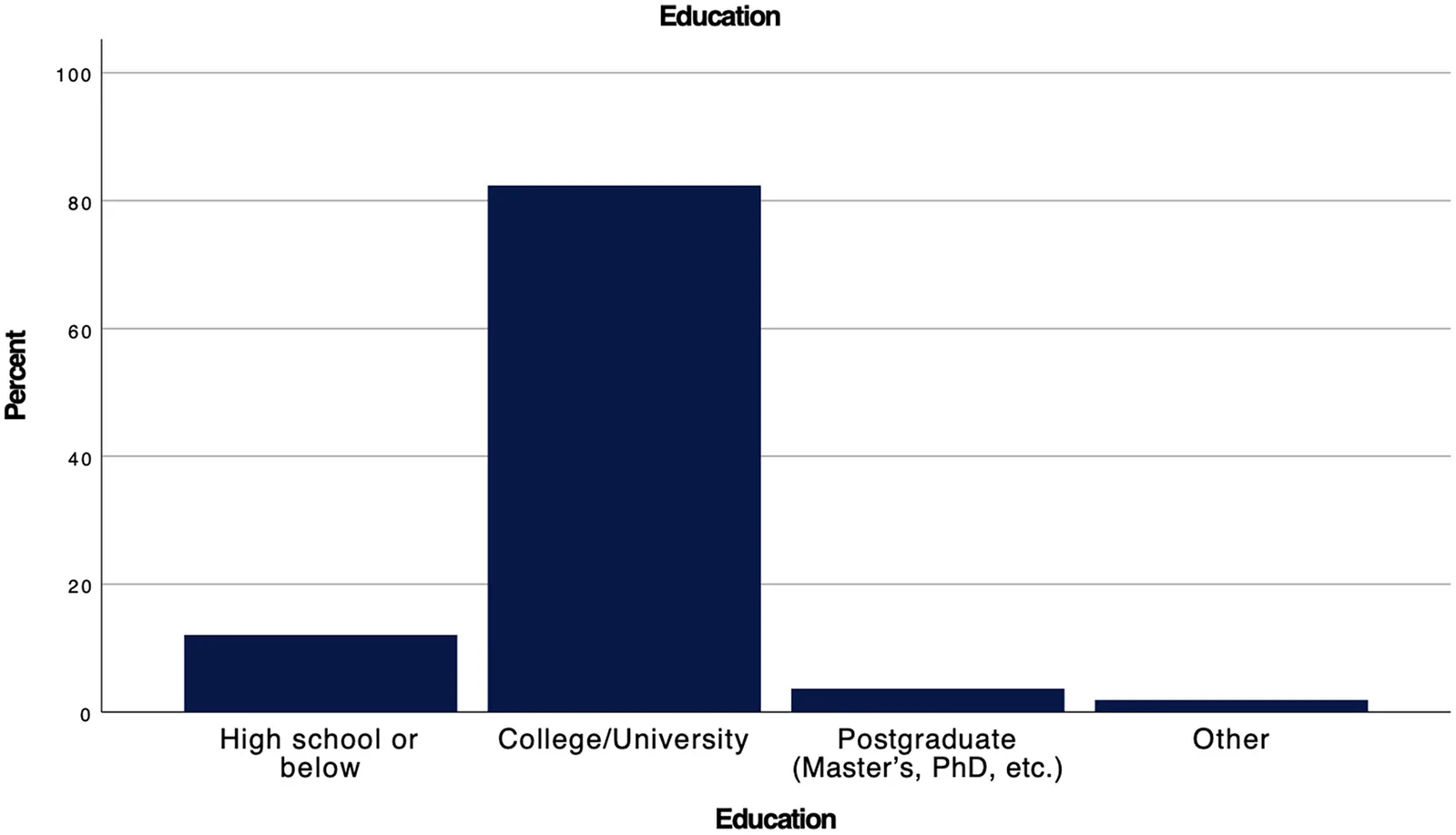
Distribution of respondents by highest level of education attained.

Fig. 1 illustrates the gender distribution, indicating that the majority of respondents are female (58.27%). Fig. 2 depicts the age distribution of the respondents, with the highest proportion belonging to the 18–24 age group (67.73%). Fig. 3 highlights the highest educational attainment, which predominantly consists of respondents holding a college or university degree (82.39%).

## 4 Experimental Design, Materials and Methods

The questionnaire was administered online using a snowball sampling approach over the period from September 2025 to January 2026. After data screening to remove incomplete or invalid responses, the final dataset consisted of 846 observations. Data analysis was conducted using SmartPLS 4. The reliability and convergent validity of the measurement model were assessed through Cronbach’s alpha (CA), composite reliability (CR), average variance extracted (AVE), and outer loadings, following the guidelines of Hair et al. (2020) [8]. Specifically, Cronbach’s alpha and composite reliability were used to evaluate the internal consistency of the constructs, with threshold values exceeding 0.7, respectively, indicating acceptable reliability. Convergent validity was examined through outer loadings and average variance extracted. Outer loadings above 0.7 suggest that the indicators adequately represent their underlying constructs, while AVE values approaching or exceeding 0.5 indicate that the constructs explain more than half of the variance of their indicators, thereby supporting convergent validity.

The survey instrument was adapted from established smart city and technology acceptance frameworks and contextualized to Viet Nam. All items were evaluated on a 5-point Likert scale ranging from 1 ("Strongly Disagree") to 5 ("Strongly Agree"), as presented in Table 2.

**Table 2:** Descriptive results of participants’ responses.

Variables	Items	N	Min	Max	Mean	Std. Deviation
**Smart Economy (CA = 0.799):** Reflects the city’s economic competitiveness, measured by innovation, entrepreneurship, economic image, productivity, labor market flexibility, international integration, and adaptability to socio-economic changes.						
**ECO1**	I perceive that the city has a high capacity for economic development, with businesses, workers, and relevant stakeholders operating effectively.	846	1	5	4.17	0.858
**ECO2**	I perceive that the city promotes creative competence and the development of new ideas among economic actors in society.	846	1	5	4.13	0.841
**ECO3**	I perceive that the economy is able to adapt flexibly to unforeseen changes, to adverse situations, and to emerging opportunities.	846	1	5	3.89	0.917
**Smart People (CA = 0.797):** Captures citizens’ competence and qualifications, emphasizing improved education, social infrastructure, creativity, and the ability to absorb and apply new technologies in daily life.						
**PEO1**	I perceive that citizens are willing to learn, absorb new ideas, and proactively propose initiatives in their daily life and work.	846	1	5	3.89	0.907
**PEO2**	I perceive that citizens are open-minded, capable of evaluating information from multiple perspectives, and tolerant of differences.	846	1	5	3.86	0.918
**PEO3**	I perceive that individuals have a deep understanding of social, economic, and political events and are able to formulate new ideas.	846	1	5	3.76	0.956
**Smart Governance (CA = 0.805):** Encompasses citizen participation in decision-making, public and social service quality enhancement, governance transparency, and strategic vision guiding urban development.						
**GOV1**	I perceive that citizens are effectively included in governmental decisions and play an active role in city administration.	846	1	5	3.75	0.969
**GOV2**	I perceive that the government cooperates effectively with citizens to better meet their needs, unify initiatives, and reduce costs.	846	1	5	3.84	0.948
**GOV3**	I perceive that information related to public management is transparent, visible, and intelligible to citizens.	846	1	5	3.93	0.918
**Smart Mobility (CA = 0.795):** Refers to applying information and communication technology (ICT) in modern, sustainable transport systems to alleviate urban congestion and enhance transit efficiency.						
**MOB1**	I perceive that the public transportation system (e.g., subways, taxis, buses) is safe and effective.	846	1	5	3.76	1.047
**MOB2**	I perceive that environmentally friendly transportation modes, such as bicycles, metro systems, or buses, are encouraged.	846	1	5	4.02	0.949
**MOB3**	I perceive that planning of roads, parking facilities, and public transport routes (e.g., buses, metro lines) in the city is convenient and effective.	846	1	5	3.72	1.054
**Smart Environment (CA = 0.841):** Focuses on environmental conditions, pollution reduction, and sustainable resource management, utilizing ICT to protect natural resources and improve air quality.						
**ENV1**	I have knowledge of environmental issues and actively seek to minimize negative environmental impacts.	846	1	5	3.96	0.903
**ENV2**	I perceive that the city implements practices to prevent and minimize pollutant emissions in order to reduce negative impacts on the environment and society.	846	1	5	3.85	0.951
**ENV3**	I perceive that the city effectively manages resources through planned management of green areas and efficient use of water and electricity.	846	1	5	3.8	0.995
**ENV4**	I perceive that urban waste is efficiently managed through recycling, renewable energy use, and waste reduction.	846	1	5	3.63	1.082
**Smart living (CA = 0.730):** Reflects citizens’ quality of life across healthcare, education, cultural facilities, housing quality, personal safety, tourism appeal, and social cohesion.						
**LIV1**	I perceive that citizens can easily access medical facilities and healthcare services (e.g., hospitals, pharmacies) in the city.	846	1	5	4.08	0.895
**LIV2**	I perceive that the city has high-quality residential infrastructure and a low level of pollution (e.g., noise, air pollution).	846	1	5	3.56	1.114
**LIV3**	I perceive that I can easily access knowledge systems, general education, libraries, and archives.	846	1	5	4.02	0.889
**Perceived Usefulness (CA = 0.831):** Measures the degree to which an individual perceives using technology in a smart city as useful, helping perform tasks faster and with better quality.						
**PU1**	I believe that implementation of smart city initiatives will bring tangible benefits to citizens’ lives.	846	1	5	4.2	0.837
**PU2**	Compared to ordinary cities, I believe that living in a smart city provides greater benefits (e.g., reduced traffic congestion, transparent and efficient public services, smart waste management).	846	1	5	4.2	0.817
**PU3**	I believe that smart cities are an improvement over the current living standards.	846	1	5	4.21	0.845
**Perceived Ease of Use (CA = 0.864):** Defined as the degree to which residents believe that participating in a smart city will be easy and effortless.						
**PEOU1**	It is easy for me to learn how to use smart city services.	846	1	5	3.98	0.868
**PEOU2**	I understand all functions of smart city services.	846	1	5	3.91	0.884
**PEOU3**	It is easy for me to use all the functions of smart city services.	846	1	5	3.87	0.924
**PEOU4**	It is easy for me to perform my daily activities via the smart city services system.	846	1	5	3.98	0.865
**Trust in new local government (CA = 0.883):** Measures citizens’ confidence in the newly restructured local administration, believing that the new government is honest and committed to residents’ interests.						
**TLG1**	I trust the new local government’s decisions regarding smart city development.	846	1	5	3.93	0.905
**TLG2**	I believe that the new local government will consider citizens’ interests in the process of smart city development.	846	1	5	4	0.874
**TLG3**	I trust the rationality of the new local government’s decisions.	846	1	5	3.88	0.902
**TLG4**	I trust that the new local government will exert its utmost effort for local development.	846	1	5	4.02	0.872
**Public participation intention (CA = 0.846):** Captures citizens’ behavioral intention to participate in smart city development, measured through their proactive engagement with smart services and adoption of new technologies.						
**PPI1**	I will obtain information about the smart city through various channels.	846	1	5	4.13	0.849
**PPI2**	I will actively participate in related activities of the smart city.	846	1	5	4.07	0.847
**PPI3**	I will encourage my friends and relatives to participate in the smart city.	846	1	5	4.09	0.849
**PPI4**	I am willing to participate in the smart city.	846	1	5	4.27	0.814

## Limitation

This study has several limitations that should be considered when interpreting the findings. First, the survey focuses primarily on cities that have been officially recognized toward smart city development in Viet Nam; therefore, the results may be more representative of large urban contexts and may not be fully generalizable to other cities or regions. In addition, the imbalance in sample sizes across the surveyed cities may affect the reliability of comparisons and introduce potential bias in multi-city analyses. Furthermore, the data was collected at a single point in time and relied on residents’ self-reported perceptions and intentions, which limits the ability to capture temporal changes or to infer actual behavioral outcomes. Finally, the uneven distribution of certain demographic groups, particularly across age categories and types of organizations, may qualify for the representativeness of the sample and should be taken into account when generalizing the results.

## Ethics Statement

This study strictly adhered to ethical principles throughout the data collection and data management processes. All participants were informed about the purpose of the study, the voluntary nature of their participation, and their right to withdraw at any time without any negative consequences. Informed consent was obtained from all respondents prior to participation. The data was used solely for academic research purposes and was absolutely confidential.

## Credit Author Statement

**Thi Thanh Hoa Phan:** Conceptualization, Methodology, Writing - reviewing and editing. **Ho Diem Chau Nguyen:** Conceptualization, Methodology, Writing original draft, Data curation, Data analysis, Writing - reviewing and editing. **Le Trung Duong:** Conceptualization, Methodology, Writing original draft, Data analysis. **Thi Yen Nhi Pham:** Methodology, Writing original draft, Data curation, Writing - reviewing and editing. **Minh Tam Bui:** Methodology, Writing original draft, Data curation, Writing - reviewing and editing. **Viet Hoang Le:** Methodology, Writing original draft, Data curation. **Van Trung Tran:** Conceptualization, Methodology, Writing original draft, Data curation, Data analysis, Writing - reviewing and editing.

## Appendix Supplementary materials

Image, application 1
